# Preemptive analgesia using selective cyclooxygenase-2 inhibitors alleviates postoperative pain in patients undergoing total knee arthroplasty: A protocol for PRISMA guided meta-analysis of randomized controlled trials

**DOI:** 10.1097/MD.0000000000024512

**Published:** 2021-02-19

**Authors:** Congcong Wang, Hongjuan Fu, Jun Wang, Fujun Huang, Xuejun Cao

**Affiliations:** a Department of the Second Joint Surgery, Weifang People's Hospital; b Department of Clinical Medicine, Weifang Medical College, Weifang; c Department of Anesthesia Surgery, Shandong Provincial Hospital, Jinan, Shandong, China.

**Keywords:** meta-analysis, pain management, preemptive analgesia, safety, selective cyclooxygenase-2 inhibitors, total knee arthroplasty

## Abstract

**Background:**

The postoperative pain associated with total knee arthroplasty (TKA) is severe for most patients. The analgesic efficacy and safety of preoperative use of selective cyclooxygenase-2 (COX-2) inhibitors for patients undergoing TKA are unclear.

**Objectives:**

We conducted a systematic review and meta-analysis to assess whether the use of selective COX-2 inhibitors before TKA decreases the postoperative pain intensity.

**Methods:**

Data sources: The PubMed, Embase, EBSCO, Web of Science, and Cochrane Controlled Register of Trials databases from inception to January 2020.

**Study eligibility criteria:**

All randomized controlled trials (RCTs) in which the intervention treatment was preoperative selective COX-2 vs placebo in patients undergoing TKA and that had at least one of the quantitative outcomes mentioned in the following section of this paper were included. Letters, review articles, case reports, editorials, animal experimental studies, and retrospective studies were excluded.

**Interventions:**

All RCTs in which the intervention treatment was preoperative selective COX-2 vs placebo in patients undergoing TKA.

**Study appraisal and synthesis methods:**

The quality of the RCTs was quantified using the Newcastle–Ottawa quality assessment scale. RevMan 5.3 software was used for the meta-analysis.

**Results:**

Six RCTs that had enrolled a total of 574 patients were included in the meta-analysis. The visual analog scale pain score at rest was significantly different between the experimental group and control group at 24 hours (*P* < .05) and 72 hours (*P* < .05) postoperatively. The experimental group exhibited a significant visual analog scale pain score during flexion at 24 hours postoperatively (*P* < .05), and it was not different at 72 hours postoperatively (*P* = .08). There was a significant difference in opioid consumption (*P* < .05), but there was no difference in the operation time (*P* = .24) or postoperative nausea/vomiting (*P* = .64) between the groups.

**Conclusion:**

The efficacy of preoperative administration of selective COX-2 inhibitors to reduce postoperative pain and opioid consumption after TKA is validated.

**Systematic review registration number:**

INPLASY202090101

## 1 Introduction

Knee osteoarthritis is a major cause of pain and disability. Knee osteoarthritis is a degenerative joint disease caused by the gradual loss of articular cartilage.^[1]^ And this disease has a high incidence among the elderly. Effective pain management is crucial to the prognosis of knee arthritis.^[2]^ Total knee arthroplasty (TKA) is effective for relief of pain and long-term improvement in quality of life among patients with knee osteoarthritis.^[3–4]^ TKA has been proven to be one of the most successful operations in the 20th century.

However, TKA is associated with considerable postoperative pain that can contribute to certain complications, delayed recovery, prolonged stiffness, chronic pain, and poor outcomes.^[5]^ Selective cyclooxygenase-2 (COX-2) inhibitors are effective analgesics for postoperative pain in patients who have undergone TKA.^[6,7]^ The preoperative use of selective COX-2 inhibitors does not increase the incidence of bleeding complications.^[8]^

Nonsteroidal anti-inflammatory drugs (NSAIDs) are usually used for postoperative analgesia. The analgesic mechanism of selective COX-2 inhibitors involves 2 main aspects.^[9]^ First, these drugs eliminate the inflammatory factors leading to pain around the peripheral damaged tissue to achieve anti-inflammatory analgesia. Second, these drugs suppress the central hypersensitivity to pain, thus reducing the patient's perception of pain. Traditional NSAIDs achieve analgesic effects by nonselectively inhibiting cyclooxygenase (COX-1 and COX-2). COX has 2 isozyme isomers. COX-1 mainly distributed in the platelets, stomach, and kidney. It catalyzes the production of physiologically needed prostaglandin E2 that regulates peripheral vascular resistance, platelet aggregation, maintains renal blood flow, and protects gastric mucosa. COX-2 is expressed by monocytes, macrophages, fibroblasts etc, in response to inflammatory stimulation, thus, it is referred to as inducible enzyme. It is one of the key enzymes that initiate inflammatory reactions and promote inflammatory response leading to tissue injury.

However, most studies focusing on the use of selective COX-2 inhibitors before TKA were small series. Therefore, we performed the present systematic review and meta-analysis of randomized controlled trials (RCTs) to assess the efficacy and safety of preoperative administration of selective COX-2 inhibitors in patients undergoing TKA. To the best of our knowledge, this is the first meta-analysis of RCTs to compare the efficacy and safety of using preoperative selective COX-2 inhibitors for pain management in patients undergoing TKA.

## 2 Materials and methods

Ethical approval and patient consent were not required because this was a systematic review and meta-analysis of previously published studies. Based on the Preferred Reporting Items for Systematic Reviews and Meta-Analyses (PRISMA), a prospective agreement on the study objectives, literature retrieval strategy, inclusion and exclusion criteria, outcome measurements, and methods of statistical analysis was established.

### 2.1 Literature search strategy

A literature search was performed in January 2020 without restriction of language, region, or publication type. The databases of MEDLINE, EMBASE, PubMed, CINAHL, Bandolier, and the Cochrane Controlled Register of Trials were searched from their inception to January 2020 by 2 of the authors (JW and HJH) using the following keywords

“TKA,” “TKR,” “total knee replacement,” “total knee arthroplasty,” “cyclooxygenase-2,” and “COX-2.” When multiple similar reports were published, the most recent report was used.

### 2.2 Inclusion and exclusion criteria

All RCTs in which the intervention treatment was preoperative selective COX-2 vs placebo in patients undergoing TKA and that had at least one of the quantitative outcomes mentioned in the following section of this paper were included. Letters, review articles, case reports, editorials, animal experimental studies, and retrospective studies were excluded.

### 2.3 Data extraction and significant outcomes

Data from the included studies were extracted and summarized independently by 2 authors (HJF and JW), and differences were resolved by consensus.

The outcome measures were

1. the visual analog scale (VAS) dynamic pain scores on the first and third days after surgery,
2. the modified numerical pain rating scale (MNPRS) score at 72 hours postoperatively,
3. morphine consumption in the first and second 24 hours postoperatively, and
4. adverse effects (nausea and vomiting).

In this study, only 2 papers adopted MNPRS score. Therefore, we obtained the raw data by contacting the author and consolidated them into VAS score.

### 2.4 Quality assessment and statistical analysis

The quality of the RCTs was quantified using the Newcastle–Ottawa quality assessment scale, which consists of three parts: patient selection, comparability of the study groups, and assessment of outcomes.

Review Manager (RevMan for Windows version 5.3; The Nordic Cochrane Centre, The Cochrane Collaboration, Copenhagen, Denmark) was used for the meta-analysis. The weighted mean difference (WMD) and 95% confidence interval (CI) were used to estimate the overall pooled effect for continuous data in each study. The relative risk and 95% CI were used to evaluate the dichotomous data. Statistical heterogeneity between studies was assessed using the chi-square test with *P* < .05. If heterogeneity was significant (*P* < .05), a random-effects model was used. If heterogeneity was not significant (*P* ≥ .05), a fixed-effects model was used.

### 2.5 Ethical review

Because all of the data used in this meta-analysis has been published, this study does not require ethical approval.

## 3 Results

### 3.1 Literature search, study characteristics, and quality assessment

**Figure 1:**
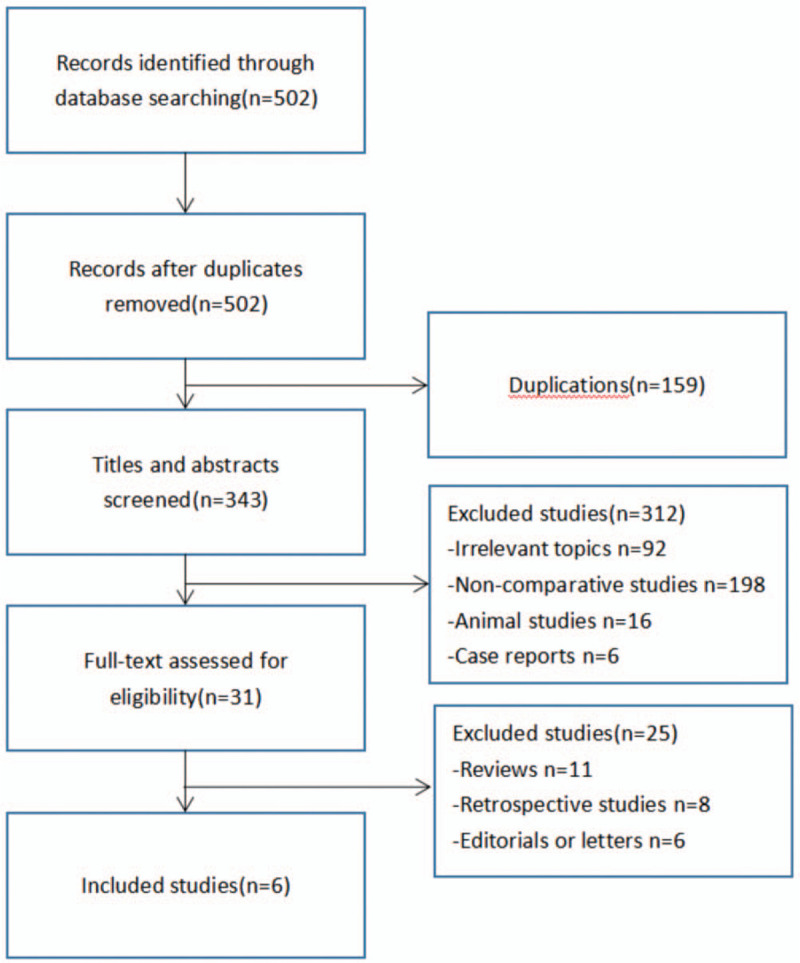
Risk of bias summary.

A detailed flow chart of the search and selection process is shown in the flow diagram (Fig. 1). Approximately 502 potentially relevant studies were tentatively identified. Finally, 6 RCTs were included in the meta-analysis.

Table 1 summarizes the baseline characteristics of the 6 eligible RCTs in the meta-analysis. The 6 studies were published from 2004 to 2018, with a total sample size of 574. In each study, the demographic characteristics of the 2 groups were similar. Agreement between the 2 reviewers was 83% for study selection and 93% for quality assessment of the RCTs.

**Table 1:** Characteristics of the included studies.

		Experimental group					Control group						
No.	Author	N	Age	Female	Duration of surgery (min)	Methods	N	Age	Female	Duration of surgery (min)	Methods	Quality score	Follow up
1	Feng 2004^[13]^	15	69.8	11	113.7	25 mg rofecoxib administered on the morning of the operation	15	64.4	13	118.5	None	5	72 h postoperatively
2	Feng 2008^[14]^	15	69.8	11	113.7	25 mg rofecoxib administered 1 hour before operation	19	64.4	13	118.5	Placebo	6	5 d postoperatively
3	Shen 2009^[10]^	30	66	18	104	200 mg celecoxib administered twice a day 3 d preoperatively	30	65	20	103	None	6	48 h postoperatively
4	Munteanu 2016^[15]^	55	66.7	44	123	120 mg etoricoxib administered 1 h before surgery	55	66.1	45	122	Placebo	7	1 y postoperatively
5	Zhongwei 2017^[11]^	62	65.2	53	—	200 mg celecoxib twice and tramadol 37.5 mg and acetaminophen 325 mg three times a day 3 days preoperatively	70	66.2	52	—	None	5	48 h postoperatively
6	Jiangfeng 2018^[12]^	105	64.8	71	107	400 mg celecoxib administered 24 hours before the operation	103	66	77	111	None	6	72 h postoperatively

Among the 6 RCTs, three studies^[10–12]^ reported the postoperative pain VAS scores, 2 studies^[13,14]^ reported the postoperative MNPRS scores, 5 studies^[10,12,13–15]^ reported opioid consumption, and 6 studies^[10–15]^ reported nausea and vomiting. The Newcastle–Ottawa quality assessment score of the 6 included studies varied from 5 to 7 stars.

### 3.2 Operating time

**Figure 2:**
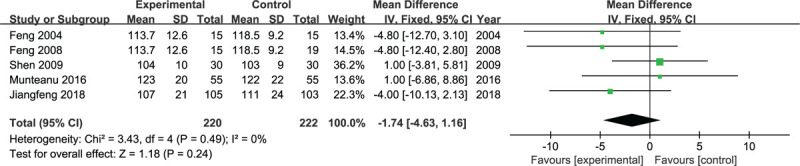
Flow diagram of study selection.

Five studies reported the operating time. Heterogeneity was present among the studies, but it was not statistically significant. Therefore, a fixed-effects model was used for the statistical analysis. There were no significant differences in the operating time between the experimental group and control group (*P* = .24, WMD = −1.74, 95% CI = −4.63 to 1.16, *I*^2^ = 0, fixed-effects model. Fig. 2).

### 3.3 Knee pain

**Figure 3:**
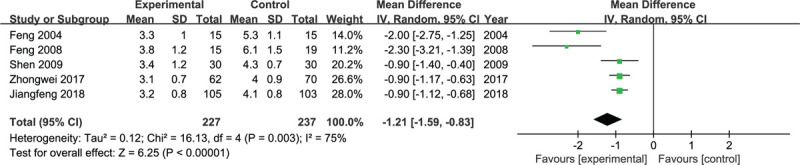
Forest plots for the meta-analysis.

**Figure 4:**
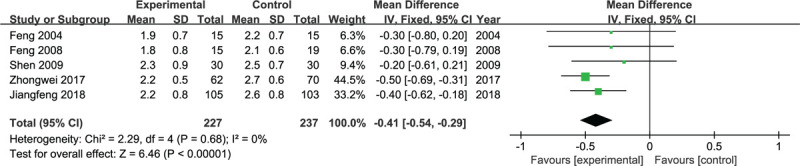
Forest plots for the meta-analysis.

**Figure 5:**
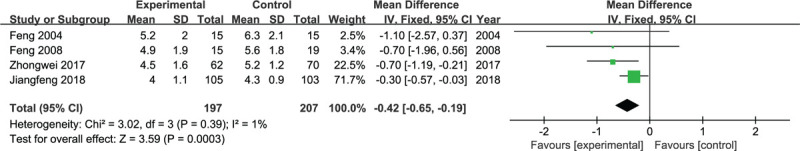
Forest plots for the meta-analysis.

**Figure 6:**
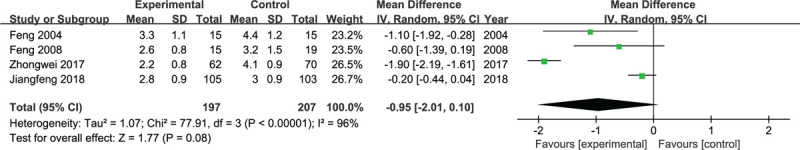
Forest plots for the meta-analysis.

The VAS pain score at rest was significantly different between the experimental group and control group at 24 hours postoperatively (*P* < .05, WMD = −1.21, 95% CI = −1.59 to −0.83, *I*^2^ = 75%, random-effects model, Fig. 3) and 72 hours postoperatively (*P* < .05, WMD = −0.41, 95% CI = −0.54 to −0.29, *I*^2^ = 0, fixed-effects model, Fig. 4). The experimental group exhibited a significant VAS pain score during flexion at 24 hours postoperatively (*P* < .05, WMD = −0.42, 95% CI = −0.65 to −0.19, *I*^2^ = 1%, fixed-effects model, Fig. 5), and it was not different at 72 hours postoperatively (*P* = .08, WMD = −0.95, 95% CI = −2.01 to 0.10, *I*^2^ = 96%, random-effects model, Fig. 6).

### 3.4 Morphine consumption

**Figure 7:**
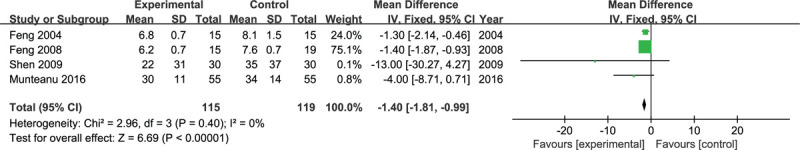
Forest plots for the meta-analysis.

**Figure 8:**
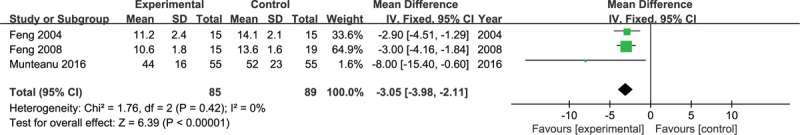
Forest plots for the meta-analysis.

**Figure 9:**
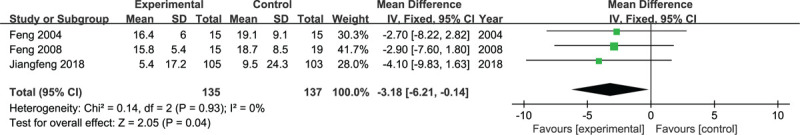
Forest plots for the meta-analysis.

Five RCTs reported the outcome of morphine consumption. The pooled results showed a significant difference in morphine consumption between the experimental and control groups at 24 hours postoperatively (*P* < .05, WMD = −1.4, 95% CI = −1.81 to −0.99, *I*^2^ = 0, fixed-effects model, Fig. 7), 48 hours postoperatively (*P* < .05, WMD = −3.05, 95% CI = −3.98 to −2.11, *I*^2^ = 0, fixed-effects model, Fig. 8), and 72 hours postoperatively (*P* < .05, WMD = −3.18, 95% CI = −6.21 to −0.14, *I*^2^ = 0, fixed-effects model, Fig. 9).

### 3.5 Nausea and vomiting

**Figure 10:**
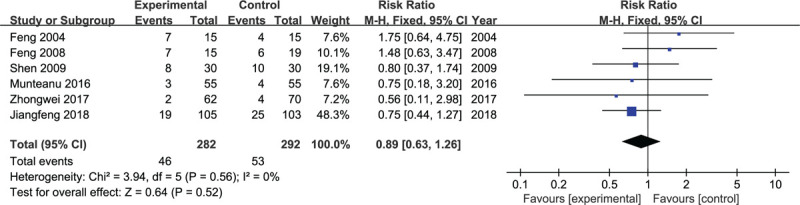
Forest plots for the meta-analysis.

All RCTs provided the outcomes of nausea and vomiting. A fixed-effects model was adopted. The risk of nausea and vomiting was not higher in the experimental than control group (*P* = .52, WMD = 0.89, 95% CI = 0.63–1.26; *P* = .52, I^2^ = 0, fixed-effects model. Fig. 10).

## 4 Discussion

To the best of our knowledge, this is the first meta-analysis of RCTs to compare the efficacy and safety of using preoperative selective COX-2 inhibitors for pain management in patients undergoing TKA. The principal finding of our study is that preoperative use of selective COX-2 inhibitors was associated with a significant reduction in knee pain and morphine consumption after TKA. In addition, no increased risk of adverse effects was found.

Knee osteoarthritis is a major cause of pain and disability, and the chronic pain associated with this condition restricts the function of the knee joints.^[16]^ Knee osteoarthritis is a degenerative joint disease caused by the gradual loss of articular cartilage.^[17–19]^ More than 50% of people aged 50 to 65 years and up to 80% of those aged >80 years report knee osteoarthritis. Effective pain management is crucial to the prognosis of knee arthritis.^[20]^

After TKA, stress such as that induced by surgical trauma and ischemia–reperfusion injury caused by use of a tourniquet can lead to the release of inflammatory mediators, making patients hypersensitive to pain. The patients’ pain threshold decreases, which affects the recovery of normal function after surgery.^[21]^ In 1 survey, about 60% of patients had severe pain and 30% had moderate pain after total knee replacement.^[22]^

**Figure 11:**
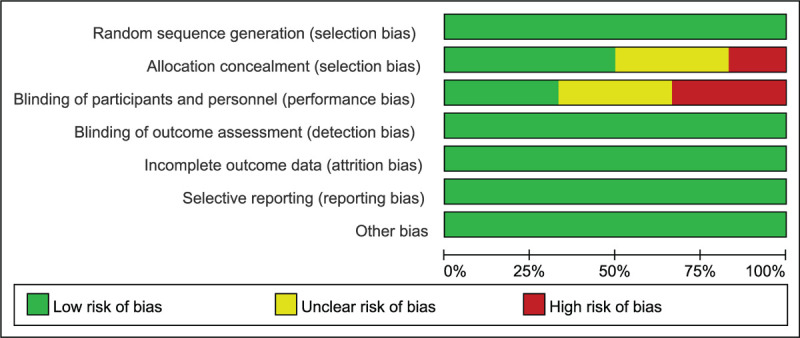
Forest plots for the meta-analysis.

**Figure 12:**
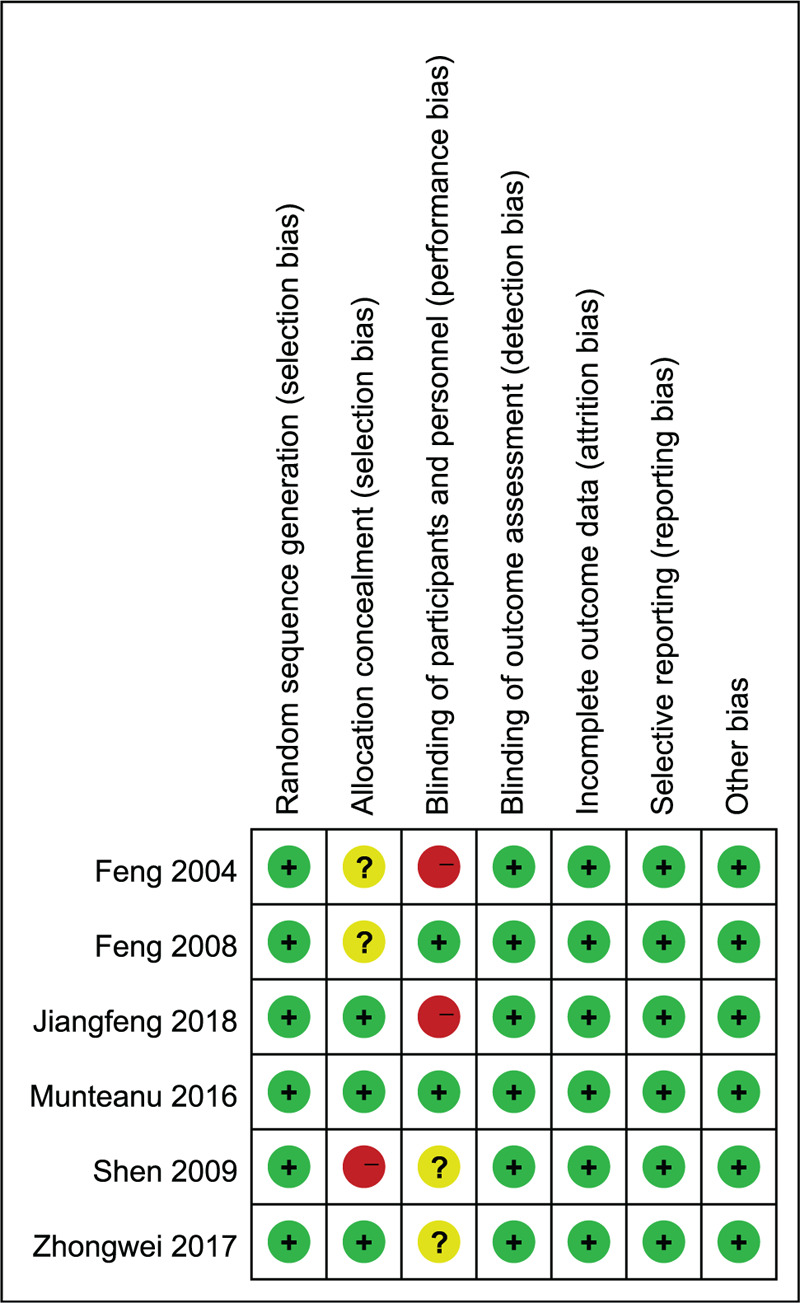
Risk of bias graph.

We identified 6 relevant studies involving 574 patients in this meta-analysis. Although we attempted to contact the authors to obtain missing data, we were unable to include every study for every outcome because of variable reports of endpoints and inconsistent definitions. Additionally, clinical heterogeneity was present among the studies. Of the 6 studies included in this analysis, 2 used rofecoxib as an analgesic, 1 used etoricoxib, and 3 used celecoxib. There was still some risk of bias in this study, as shown in Figures 11 and 12.

Several studies^[23–28]^ have shown that the use of selective COX-2 inhibitors in the perioperative period can have a good effect on pain management after total knee replacement. However, whether preoperative or postoperative use of selective COX-2 inhibitors is more effective remains unclear. Pain scores are typically the primary outcome measured in clinical pain studies. In the present study, the resting VAS score reached statistical significance during the first 72 hours after TKA in the preemptive analgesia group, and the VAS score during flexion reached statistical significance during the first 24 hours after TKA in the preemptive analgesia group. The MNPRS score reached statistical significance during the first 48 hours after TKA in the preemptive analgesia group both at rest and during flexion. Effective analgesia not only reduces the stress response to the operation and improves the patients’ anxiety state but also helps patients to carry out functional exercise as soon as possible. Clinicians should strive to restore knee range of motion and prevent complications such as deep vein thrombosis. Selective COX-2 inhibitors can effectively eliminate pain-inducing factors at their source and reduce the severity of pain with lower opioid drug use. This meta-analysis showed that compared with the control group, the use of selective COX-2 inhibitors before surgery reduced the amount of morphine used at 72 hours after surgery. However, in terms of adverse reactions, the incidence of nausea and vomiting was not significantly different between the 2 groups. Two studies^[13,14]^ have reported the efficacy of COX-2 inhibitors on interleukin-6 production. The experimental groups showed 30% lower blood levels of interleukin-6 compared with controls at 6, 12, and 48 hours postoperatively in patients who had undergone TKA.

The types of selective COX-2 inhibitors, preoperative administration time, administration route, COX-2 dosage, and use of postoperative analgesic pump drugs included in this study were not consistent, which may affect the strength of our conclusions. Because the methods were not standardized among the six studies, a high-quality, large-sample, multicenter RCT is needed to verify the conclusions of this meta-analysis.

## 5 Conclusion

In summary, this meta-analysis confirmed the efficacy of preoperative use of selective COX-2 inhibitors in reducing pain and opioid consumption after TKA without increasing the incidence of nausea and vomiting.

## Acknowledgments

We thank Angela Morben, DVM, ELS, from Liwen Bianji, Edanz Editing China (www.liwenbianji.cn/ac), for editing the English text of a draft of this manuscript.

## Author contributions

**Conceptualization:** Congcong Wang.

**Data curation**: Hongjuan Fu, Jun Wang.

**Funding acquisition:** Xuejun Cao.

**Methodology**: Jun Wang, Fujun Huang.

**Project administration:** Congcong Wang

**Supervision**: Xuejun Cao.

**Writing – original draft:** Fujun Huang.

**Writing – review & editing:** Congcong Wang.
